# A robot localization proposal for the RobotAtFactory 4.0: A novel robotics competition within the Industry 4.0 concept

**DOI:** 10.3389/frobt.2022.1023590

**Published:** 2022-11-15

**Authors:** João Braun, Alexandre O. Júnior, Guido Berger, Vítor H. Pinto, Inês N. Soares, Ana I. Pereira, José Lima, Paulo Costa

**Affiliations:** ^1^ Research Centre in Digitalization and Intelligent Robotics (CeDRI), Instituto Politécnico de Bragança, Bragança, Portugal; ^2^ Laboratory for Sustainability and Technology in Mountain Regions (SusTEC), Instituto Politécnico de Bragança, Bragança, Portugal; ^3^ INESC Technology and Science, Centre in Robotics in Industry and Intelligent Systems, Porto, Portugal; ^4^ Department of Electrical and Computer Engineering, FEUP—Faculty of Engineering of the University of Porto, Porto, Portugal; ^5^ SYSTEC (DIGI2)—Research Center for Systems and Technologies, Laboratory for Digital and Intelligent Industry, Porto, Portugal

**Keywords:** autonomous mobile robot, indoor localization, Industry 4.0, robotics competition, fiducial markers, Education 4.0, STEM education, robotics in education

## Abstract

Robotic competitions are an excellent way to promote innovative solutions for the current industries’ challenges and entrepreneurial spirit, acquire technical and transversal skills through active teaching, and promote this area to the public. In other words, since robotics is a multidisciplinary field, its competitions address several knowledge topics, especially in the STEM (Science, Technology, Engineering, and Mathematics) category, that are shared among the students and researchers, driving further technology and science. A new competition encompassed in the Portuguese Robotics Open was created according to the Industry 4.0 concept in the production chain. In this competition, RobotAtFactory 4.0, a shop floor, is used to mimic a fully automated industrial logistics warehouse and the challenges it brings. Autonomous Mobile Robots (AMRs) must be used to operate without supervision and perform the tasks that the warehouse requests. There are different types of boxes which dictate their partial and definitive destinations. In this reasoning, AMRs should identify each and transport them to their destinations. This paper describes an approach to the indoor localization system for the competition based on the Extended Kalman Filter (EKF) and ArUco markers. Different innovation methods for the obtained observations were tested and compared in the EKF. A real robot was designed and assembled to act as a test bed for the localization system’s validation. Thus, the approach was validated in the real scenario using a factory floor with the official specifications provided by the competition organization.

## 1 Introduction

**FIGURE 1:**
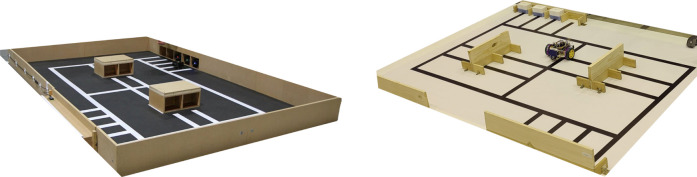
RobotAtFactory original field (left). RobotAtFactory Lite field (right).

The RobotAtFactory competition was introduced in the Portuguese Robotics Open in 2010 and has been held yearly in this national event that brings several national and international robotics competitors. This competition challenges participants to develop autonomous mobile robots (AMRs) that must transport boxes in a warehouse environment, reproducing an industrial problem scenario on a small scale. The first version of this competition had the field shown in the left picture of Figure 1. This classic competition consisted of transporting five boxes representing materials through the factory until they reached the outgoing warehouse. Moreover, the contest was composed of three 10 min rounds, where the degree of difficulty increased progressively. This increase in difficulty was translated to different types of boxes that had intermediate objectives that needed to be accomplished before being delivered to the exit of the warehouse. These partial objectives were being delivered and processed by one or two machines in series in the second and third rounds, respectively. In order to differentiate each box type, a colour LED was placed on the front side of the box. The box that needed to be processed once used the green LED, two processes, a red LED, and a blue LED for no processes requirements. The robot should be able to sense the colour LED and manage the algorithm according to it. Finally, the robot should also have a mechanical structure like a forklift to pick up the boxes.

Meanwhile, a simplified version was introduced in 2019, the RobotAtFactory Lite competition (whose field is shown in the right picture of Figure 1). Several modifications were made to simplify the complexity and difficulty of the classic RAF competition. First, the identification of the box type (Green, Red or Blue) is made by reading the RFID tag placed inside the box by the robot. Also, the box was simplified and lighter, with a metallic plate on the front side so that the robot could pick the part with a magnet, avoiding the complexity of the forklift. Moreover, in the Lite version, only four boxes are needed to be transported at a given round, one less than in the classic version. These modifications were made to make the competition less demanding and expensive, and consequently, the robot’s complexity. This competition was a success since several teams were participating. In fact, one can The mechanical difficulty of the original RobotAtFactory pushed this new Lite version competition.

**FIGURE 2:**
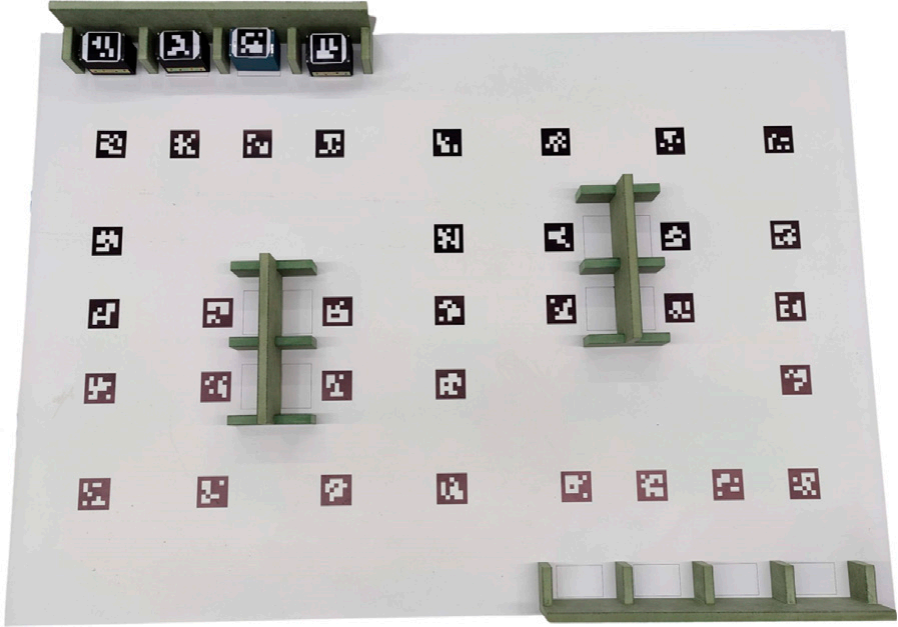
RAF competition field.

The high impact that RobotAtFactory Lite had by the team’s feedback made the competition’s creators update the original RobotAtFactory. The boxes are now the same. The field is also the same size as the RobotAtFactory Lite, and the warehouses and machines supports are the same, facilitating the organization. The new competition called RobotAtFactory 4.0 (RAF), is presented in Figure 2. An important difference from RobotAtFactory Lite is the floor lines that were replaced by ArUco markers.

The organization provides the ID and the absolute pose (position and orientation relative to the global reference frame) for each ArUco marker that the teams can use to localize the robot. The ArUco markers placed on the top of the boxes will be further used by the organization to track the objects in real time and, thus, develop an automatic referee. Another difference from the RobotAtFactory Lite is that the RFID is nonexistent, obliging the robot to request the referee’s server, using UDP protocol, for each part type to optimize the scheduling of the tasks. Inaugurated in the 2022 Portuguese Robotics Open edition, this competition version differentiates from the previous by updating it to the Industry 4.0 (I4.0) paradigm.

In other words, compared to RobotAtFactory, RAF focuses more on robot localization, path and trajectory planning, and Internet of Things (IoT) problems instead of mechanical ones. These challenges are the ones that the I4.0 scenario faces, where AMRs operate without supervision alongside personnel. In this way, RAF tries to mimic these challenges in a competition scenario where it represents the automated warehouse that links other sections of an automated production chain.

Moreover, the organization provides a simulation scene representing the official competition environment in the Simtwo simulator^1^, a realistic 3D rigid-body dynamics simulator. Besides the environment scene having all the competition aspects, it also has an architecture example of an AMR with an RGB camera and other sensors for localization. This way, a description of the simulator with the image acquisition and ZMQ^2^ communication will be presented. Also, by sending images by ZMQ, the simulator allows for development in a hardware-in-the-loop simulation approach, increasing the simulation’s effectiveness. This way, it is possible to implement an architecture similar to the real scenario. For example, a Raspberry Pi can be inserted into the loop simulation, adding the hardware’s computational limitations to process the images and control the robot in the simulator.

Therefore, this paper will describe the new RAF competition by addressing the challenges that must be faced by researchers, engineers, and students with a focus on robot localization. This focus happens because there are no publications on this matter for this competition, which is one of the crucial challenges in the I4.0 scope. Thus, a localization system that combines an Extended Kalman Filter (EKF) and the Mahalanobis filter with observations of ArUco tags is proposed and validated through comparisons with a developed ground truth system. Moreover, different methods of innovation for the acquired observations in the EKF were tested and compared. Therefore, the proposed system is presented as a reliable solution for mobile robots’ localization in the RAF competition scenario and as a basis for other scenarios involving the use of fiducial markers. The kidnapped robot problem will be addressed for system validation since it is a common problem in autonomous robotics and presents a higher resolution complexity than position tracking and global localization problems.

The remainder of the paper is structured as follows. Section 2 presents the related work where the fiducial markers for localization and other similar robotic competitions are addressed. Moreover, the RAF rules are also described in this section. Then, Section 3 shows the real robot architecture with subsections that present the ROS-SimTwo framework and the method used to obtain the robot’s pose using ArUco tags. Along Section 4, the proposed robot’s localization system is presented, emphasizing the EKF and the filters used. In turn, Section 5 discusses the results comparing the different approaches for localization, validating it using the kidnapped robot problem case study. Finally, Section 6 concludes the paper and presents future works.

## 2 Related work

AMRs can move freely in their workspace without human interference. These types of autonomous robots require a precise localization system to be able to traverse the floor plan to perform their tasks. Apart from commonly implemented methods for localization, such as Simultaneous Localization and Mapping (SLAM) and Global Positioning System (GPS), fiducial markers can be used singly or to support other localization systems.

### 2.1 Fiducial markers for localization

Fiducial markers have specific patterns and work as landmarks for pose estimation, requiring an imaging system to identify them. They are designed with a well-characterized size and shape, and their distinctive visual features are essential to improve the accuracy and robustness of a robot's localization system (Kalaitzakis et al., 2021a). Furthermore, their patterns often embed specific encoding to avoid misdetections.

According to the study provided by Kalaitzakis et al. (2021a), fiducial markers are either circular or square-shaped and monochromatic, with most squared markers being based on ARToolKit, an open-source tracking system originally described for video-based augmented reality conferencing systems Kato and Billinghurst (1999). ARTag Fiala (2005), AprilTag Olson (2011), ArUco Garrido-Jurado et al. (2014), and STag Benligiray et al. (2019) correspond to widely used packages of fiducial markers. Examples of circular markers include CCC (concentric contrasting circles) Gatrell et al. (1992), CCTag Calvet et al. (2016), and TRIP López-de Ipiña et al. (2002).

STag differentiates from other squared markers, which feature a circular pattern in the centre. ArUco builds on both ARTag and ARToolKit frameworks and provides user-configurable libraries. Fiducial markers have been reported multiple times for pose estimation in robotics applications, that is, to determine the precise position and orientation of the vehicle in the environment.

An indoor localization system is proposed by combining the observations of ARTags scattered around the environment and the adaptative Monte Carlo particle filter that uses a LIDAR sensor and the robot’s odometry in de Oliveira Júnior et al. (2021). The research displays an effective strategy for AMR localization, especially when considering the global localization problem. ArUco markers were used in Hayat et al. (2019) for the development of Tarantula, a legged-wheeled robot designed to inspect drainage systems. They served a double purpose: identify the kinematic parameters of the robot and test its trajectory tracking.

Xu et al. (2021) propose a strategy for underwater visual navigation based on multiple ArUco markers. Transformation matrices are used to derive the camera’s position attached to the robot concerning different markers, whose positions are fed to a noise model due to the noise associated with underwater imaging. The noise model provides estimation with a single marker, which is combined in an optimal algorithm with the predicted pose from multiple markers. ArUco markers are used in Muñoz-Salinas et al. (2018) for localization and mapping. The proposed methodology generates an initial pose graph from the observed markers, which is then refined by distributing the errors, and the poses are finally optimized.

In this paper, a sensor fusion approach with ArUcos fiducial markers and the odometry of a mobile robot is used to develop a localization system for the RAF competition scenario being evaluated and compared with a ground truth system.

### 2.2 Robotic competitions

Robot competitions are an excellent way to foment research and attract students to technological areas by introducing new technologies, teamwork Kandlhofer and Steinbauer (2016) and even developing solutions to real challenges in the industry. Competitions also search for different solutions to a proposed technological challenge Nugent et al. (2016).

There are a vast number of robotic competitions around the world, such as the RoboCupSoccer competition, founded in 1997 in Nogaya, Japan. It consists of a competition where teams of robots compete in a soccer match proposed by Mackworth (1993). The RoboCupSoccer are held in five leagues: Middle Size League, Small Size League, humanoid, four-legged, and simulation Soetens et al. (2014); Weitzenfeld et al. (2014). Over the years, these competitions have contributed to scientific advancements in areas such as the robust design of mechatronic systems, localization, sensor-fusion, tracking, world modelling, and distributed multi-agent coordination Soetens et al. (2014).

According to Burkhard et al. (2002), the ultimate goal of the RoboCup initiative is, by the mid-21st century, a team of fully autonomous humanoid robots soccer players shall win a soccer game, complying with the official rule of FIFA, against the winner of the most recent World Cup. The DARPA Robotics Challenge is another competition funded by the US Defense Advanced Research Projects Agency where semi-autonomous ground robots should do complex tasks in dangerous, degraded and human-engineered environments.

Another important competition in autonomous robots is the Swarmathon, where the challenge is to program robot swarms to search for, pick up and drop off resources in collection areas Lu et al. (2018). Swarmathon tries to simulate the terrestrial environment of Mars so that participants can contribute to collecting resources on the surface of the red planet. As a result, the competition has encouraged several students to venture into the robotics field Ackerman et al. (2018).

A competition that describes the essential components for the Factories of Future (FoF) is RoboCup@Work, which was established in 2012 Kraetzschmar et al. (2015). This competition uses mobile robots equipped with manipulators in scenarios representing complex and challenging industrial environments to detect, manipulate and transport objects, evaluating the autonomous and adaptive capacity of the robots Carstensen et al. (2015). Some of the challenges to be faced by the teams to get good results in this competition are related to perception, path planning and motion planning, mobile manipulation, planning and scheduling, learning and adaptability, and probabilistic modelling Kraetzschmar et al. (2015). Mobile robotic competitions are also a great way for motivating students and professionals for the FoF fields, as mentioned by Marouani (2022) and Brancalião et al. (2022).

There is considerable concern about the I4.0 concept teaching. Papers such as Tosello et al. (2019); Verner et al. (2020) focus on using robotics to teach and train students for the I4.0 era. This concern is also latent in several publications that mention the incorporation of I4.0 in the multiple levels of education, such as the works of Akgunduz and Mesutoglu (2021), Mystakidis et al. (2021) and Mystakidis and Christopoulos (2022). These agree that, in addition to providing students with increased capabilities, the use of project-based learning considering the STEAM (Science, Technology, Engineering, Arts, and Mathematics) fields and I4.0 also help teachers obtain greater knowledge about the area which is presented as fundamental. Even during the COVID-19 pandemic, some authors understood that the I4.0 concepts are so crucial that they created online materials to teach these subjects, as mentioned by Benis et al. (2021), Jain and Jain (2021) and Jena et al. (2022).

STEAM education is a popular pedagogical approach for enhancing the students’ creativity and problem-solving skills, increasing their interest in these areas Perignat and Katz-Buonincontro (2019). Since robotics addresses multidisciplinary areas, it plays an essential role in the STEAM concept. Thus, robotics competitions are an excellent tool that teachers might use to address the different topics, as stated by Weinberg et al. (2001), that divides the robotics concepts into four main areas, namely Computer Science, Mechanical Engineering, Industrial Engineering, and Electrical and Computer Engineering. So, Robotics is an exciting area to address the science, technology, engineering, and mathematics (STEM) teaching.

### 2.3 Rules RobotAtFactory 4.0

Since the advent of the fourth industrial revolution concept, new challenges have emerged. In this way, researchers and engineers must propose solutions to address these challenges, implement the I4.0 concept in real life, or improve the already implemented solutions. One of the ways to foster solutions for these challenges is through robotic competitions that mimic the industry environment with its problems. In addition, these competitions encourage students to study several areas of knowledge that belong to robotics, including STEM.

**FIGURE 3:**
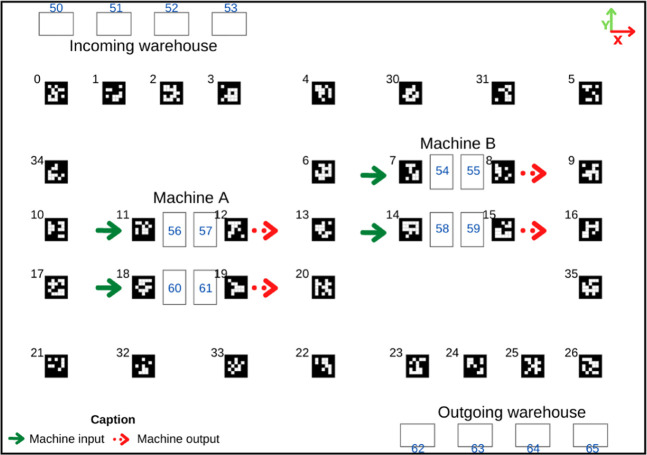
RAF official competition floor illustration Braun et al. (2022).

As previously mentioned in Section 1, the RAF competition tries to mimic a fully automated industry-warehouse environment with boxes organized by AMRs. Thus, the robots must self-localize and navigate whilst avoiding collisions. In addition, they should identify the types of boxes and collect and transport them to their respective spots in the shorted time possible. The official competition floor is displayed in Figure 3.

The boxes that the AMRs must organize are displaced in the four blank rectangles represented in Figure 3 where it is indicated “incoming warehouse.” There are three types of boxes, the first one, called the raw box, must be transported to machine type A, soon after machine type B, and then finally to the outgoing warehouse. The second type, the intermediate box, must be moved to machine type B and later the exit. Lastly, the third one, called the final box, must be displaced directly to the outgoing warehouse. As already mentioned in Section 1, the RAF competition was updated from the classic competition RobotAtFactory Costa et al. (2016) to address the I4.0 concept. Thus, the competition floor and the walls of the input and output warehouses are filled with fiducial markers that the competitors can use for localization Kalaitzakis et al. (2021b); Roos-Hoefgeest et al. (2021). Figure 3 displays the markers’ IDs and their respective positions alongside the world’s coordinate frame.

The artificial markers displaced in the competition environment displayed in Figure 3 use the ArUco 5 × 5 marker pattern of codification. There is a restriction to the size of the AMR, which must fit into a cube of 30 cm × 30 cm × 30 cm. There is neither a restriction on the number of robots a team can use simultaneously nor which type of localization system the robots can have. The robots must be completely autonomous and cannot establish any communication with an external system despite the one provided by the organization. This system is a task assignment server that the robot can communicate with to request info about which boxes are in the incoming warehouse and if the slots in the machines are occupied. The competition is divided into three rounds with increasing difficulty, including raw and intermediate boxes. To profoundly understand the competition characteristics and rules, the reader is referred to Costa et al. (2022).

## 3 Robot architecture

**FIGURE 4:**
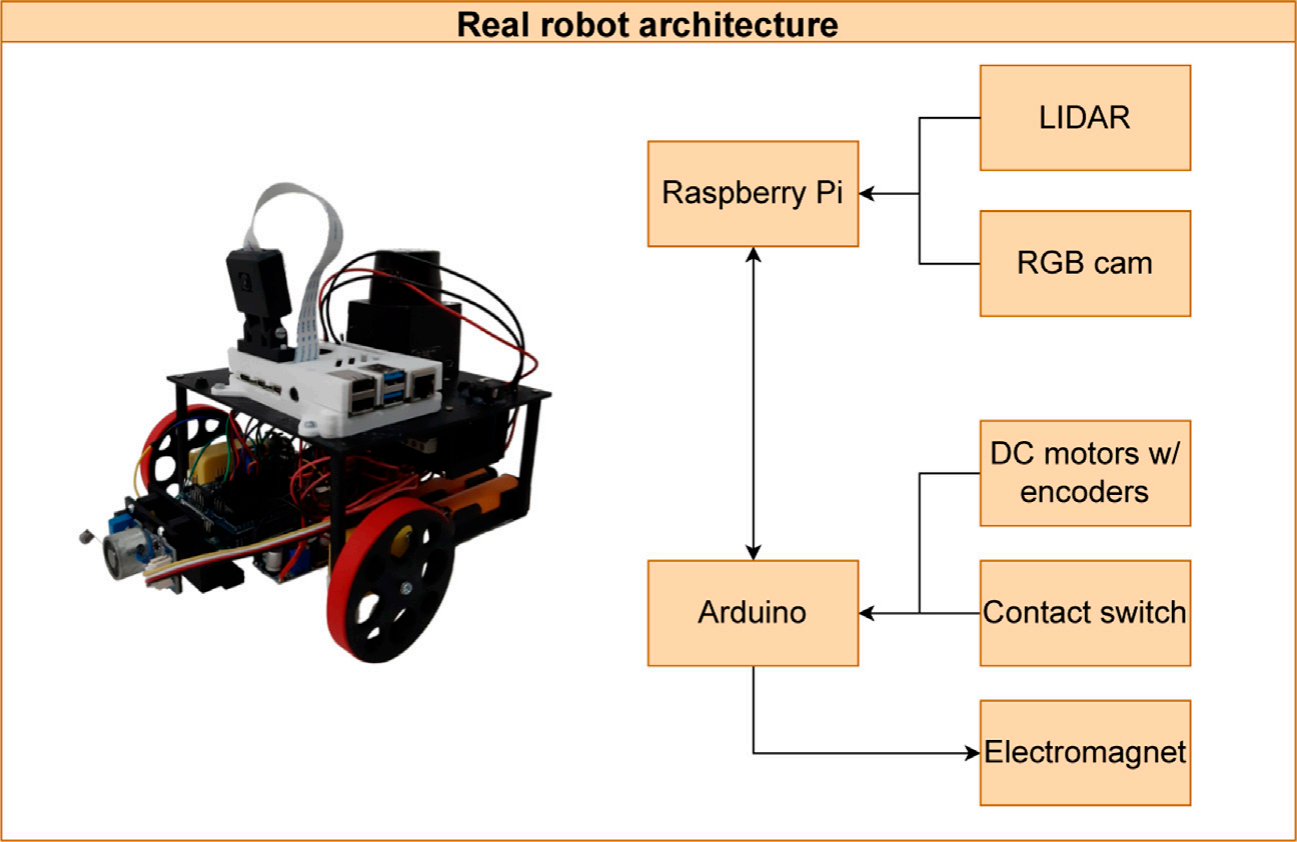
AMR and its components Braun et al. (2022).

The AMR used in the contest is a differential-drive robot with several components to operate without supervision on the factory floor. Its components can be seen in Figure 4.

As it is possible to see, the AMR has a Raspberry Pi 4B to perform the high-level control of the robot, which constitutes several processes for the robot to become autonomous, starting from cognition to path and trajectory planning, configuration, odometry integration, localization, and mapping. In addition, the robot has an RGB camera and a LIDAR scanner that is used for localization purposes. However, the latter is going to be used just in future works. Finally, it has one Arduino Uno to perform the low-level control of the robot, which constitutes the generation of the control cycle, the execution of the speed controllers of the DC motors, odometry, the reading of a contact switch sensor, and the actuation of an electromagnet.

**FIGURE 5:**
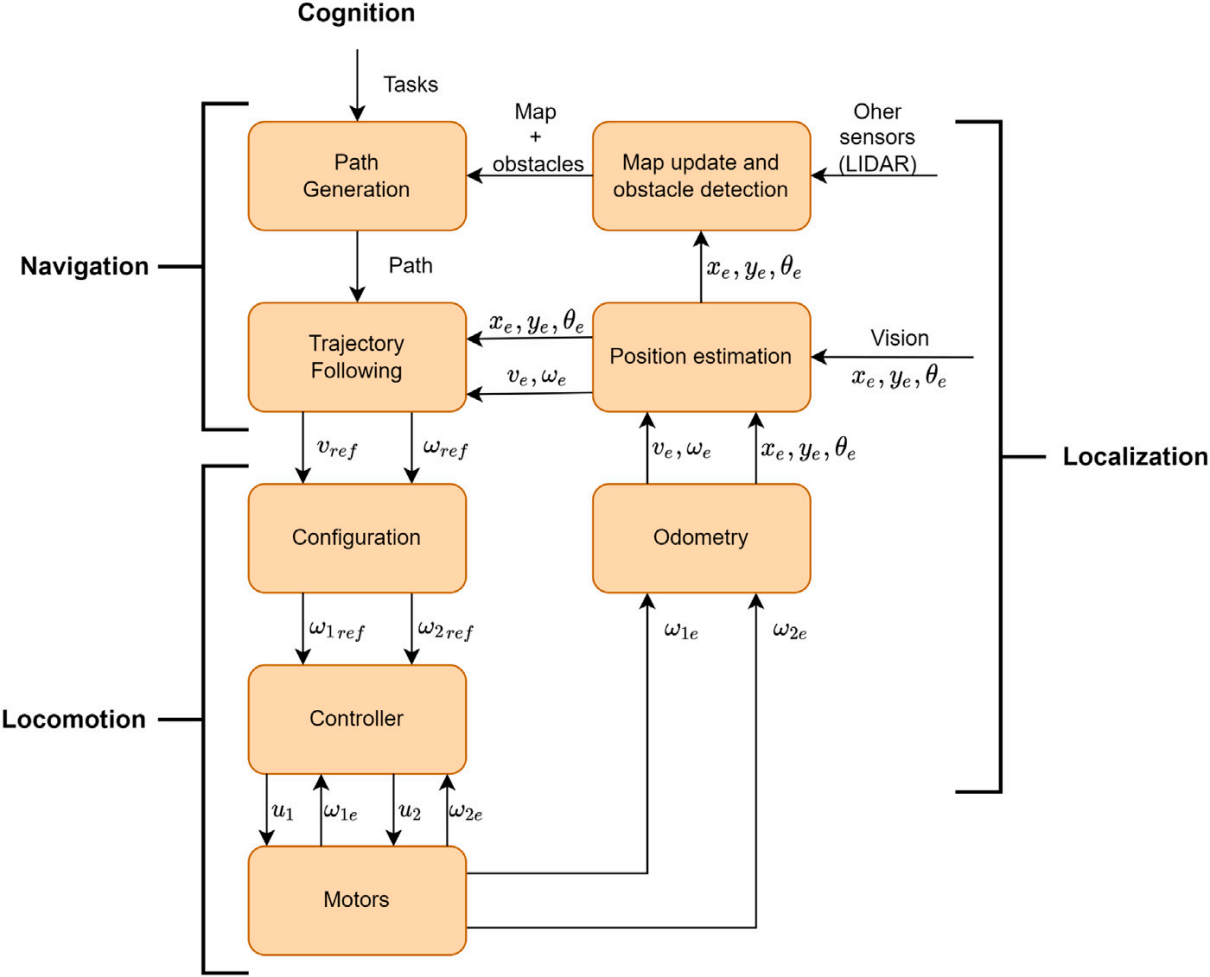
Differential-drive AMR’s architecture flowchart. *x*_*e*_, *y*_*e*_, and *θ*_*e*_ are respectively the estimated position and orientation of the robot; *v*_*ref*_ and *ω*_*ref*_ are the linear and angular reference speeds; *ω*_*1ref*_ and *ω*_*2ref*_ are the reference speeds for the right and left wheels; *u*_1_ and *u*_2_ are voltages applied to the motors; *ω*_1*e*_ and *ω*_2*e*_ are the angular estimated speeds; *v*_*e*_ and *ω*_*e*_ are the linear and angular estimated speeds.

**FIGURE 6:**
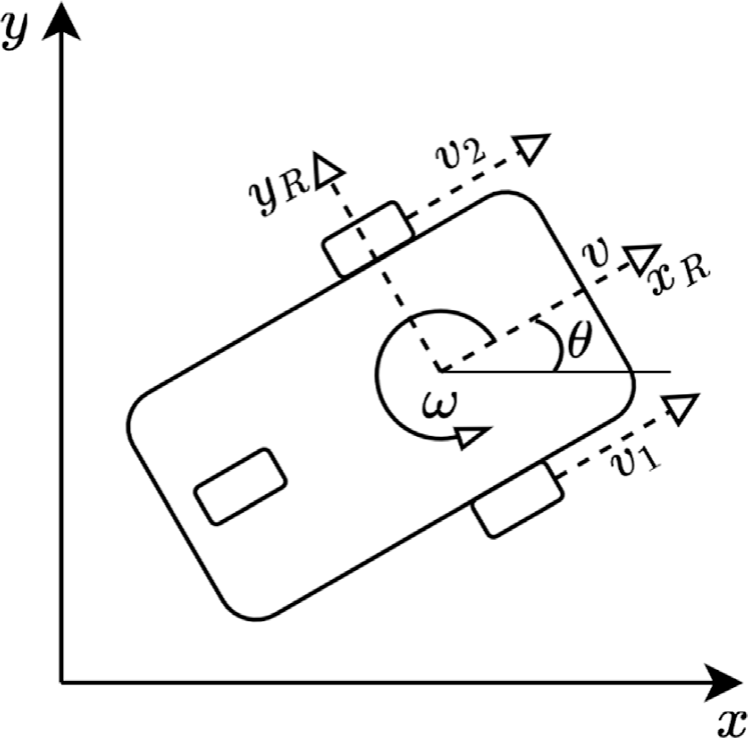
Differential-drive mobile robot kinematic model.

All these operations are processes that the AMR must accomplish to become autonomous, and they are displayed in Figure 5 in a flowchart format.As the flowchart demonstrates, an AMR comprises several processes: cognition, navigation, locomotion, and localization. Each process includes several subprocesses. In this way, first comes the cognition process that is omitted and considered as an input in this flowchart. This process is abstract and represents the robot’s AI that can also generate tasks it must accomplish to achieve its goals. After a task is generated, the entire system starts to operate. Therefore, the path generation block generates a path for the robot to follow to accomplish the given task. This block generates the path taking into account a pre-generated map. The data within constitutes the environment, such as obstacles, their positions and dimensions, and the dimension of the map. After the path’s generation, the trajectory planning is computed, which constitutes how the robot will follow the generated path. In other words, it will calculate the new robot’s speed states with the current robot’s states (pose and speed). It is important to note that it was not developed a collision avoidance system in this architecture since there are no dynamic obstacles, the fixed ones can be mapped, and only one AMR was used in the competition. Afterwards, the locomotion process starts. In this process, the speeds’ states are translated to the robot’s driving architecture, which is differential. Thus, *v*_*ref*_ and *w*_*ref*_ are transformed by the robot’s kinematic drive model to *ω*_*1ref*_ and *ω*_*2ref*_, which are the motors’ speeds in the configuration block. Figure 6 displays a differential-drive mobile robot sketch with its speeds’ states.

The global reference frame is represented by [*x*, *y*] and [*x*_*R*_, *y*_*R*_] is the robot’s body frame. The left wheel’s linear speed is described by *v*_2_ = *ω*_2_ *r* considering that *r* is the wheel’s radius and that *v*_1_ is the equivalent of the former but for the right wheel. The robot’s linear speed is represented by *v*, *ω* is the robot’s steering or angular speed, and *θ* is the robot’s orientation concerning the global frame. Thus, Eq. 1 displays the kinematic model derived from this drive architecture (for *θ* = 0), where *d* represents the distance between the midpoint of the wheels.

$$\left\{\begin{array}{c} v=v_{x}=\frac{v_{1}+v_{2}}{2}\;\\ \omega =\frac{r}{d}\left(\omega_{1}-\omega_{2}\right)\; \end{array}\right.$$ (1)

Finally, the reader is referred to Malu and Majumdar (2014) for a complete review of this driving architecture. Subsequently, the wheels’ must be controlled individually so that the robot can achieve and maintain its speed states given by the trajectory following block. In this sense, the motors and the controller blocks communicate to perform the speed control of both motors. The controller block receives the estimated motors’ speeds read by the encoders, which in turn, computes the necessary voltage signals for the motors to achieve the reference speeds produced by the trajectory following block. Then, the odometry block performs the dead reckoning by estimating the robot’s following pose states using its previous pose and speed states. The next block, position estimation, approximates the robot’s pose using the odometry and exteroceptive data. In other words, this block contains a position filter that performs this estimation. This block, however, will be thoroughly explained in Section 4. Thus, after the pose is estimated in the current control cycle, the map is updated with newer environment data estimated with the aid of the new robot’s pose estimate and other exteroceptive sensors’ data.

**FIGURE 7:**
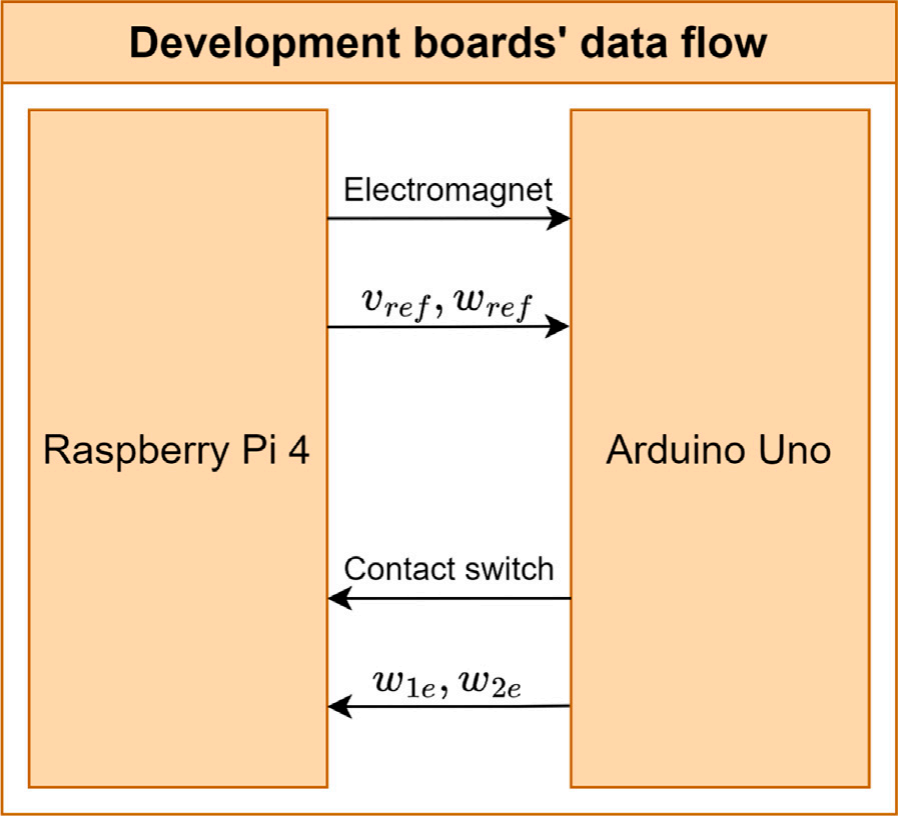
Data flow between the two development boards of the AMR.

As mentioned at the beginning of this section, the Raspberry Pi 4B performs the high-level control of the robot, whereas the Arduino Uno performs the low-level control of it. Thus, considering the perspective from Figure 5, the cognition, navigation, and localization processes occur in the Raspberry Pi 4B while the locomotion process happens in the Arduino Uno. Finally, Figure 7 displays the data flow between the two development boards.

As one can see, the Raspberry Pi 4B sends the robot’s speed states and the electromagnet actuation whereas the Arduino Uno sends back the contact switch readings and the wheels’ estimated speeds read by the encoder.

### 3.1 Robot architecture integrated within the ROS middleware

The organizers of the RobotAtFactory Lite and RAF competitions provide the participants with virtual scenarios of these competitions through the SimTwo robotics simulator, giving the participants a practical and realistic way to validate their software solutions and methodologies during their development.

Other software tools such as the middleware Robot Operating System (ROS) act as facilitators in the development of robotics solutions, standardizing message exchange systems and offering a vast set of libraries and packages with algorithms that address solutions to common problems in robotics and make it easy the integration of peripherals, actuators and sensors to these approaches. These factors and the popularization of ROS allowed its compatibility with several robotic simulators such as Gazebo, CoppeliaSim, AirSim, Webots, MORSE, and others, allowing applications developed in ROS in these simulators to be integrated without significant adaptations to real robots.

Therefore, an integration framework was developed between the SimTwo simulator and the ROS middleware to develop solutions compatible with the simulator and mobile robots integrated with ROS. The communication between the ROS and the SimTwo simulator demands the UDP and ZMQ communication protocols. The UDP communication protocol sends and receives data packets from most peripherals of the robot modelled in software, i.e., encoders, electromagnet, LiDAR, and others.

**FIGURE 8:**
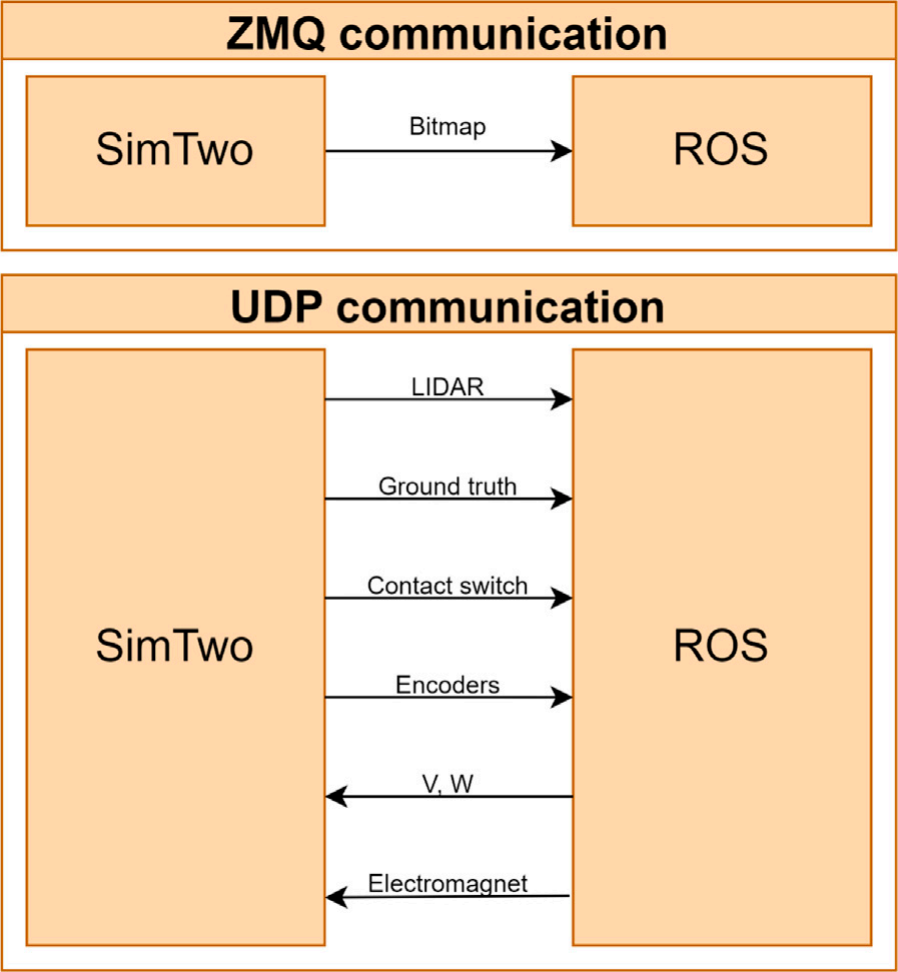
SimTwo-ROS framework Braun et al. (2022).

As shown in Figure 8, part of the information sent by the UDP protocol is encoded in a string with labels for each value and then subsequently decoded on the ROS side. On each SimTwo control cycle, the distance detection values provided by the LiDAR sensor, ground truth, contact switch, and encoders are sent. Subsequently, the ROS framework performs robot speed state calculations and sends them *via* UDP datagram. In addition, the on/off commands for the electromagnet are also sent *via* ROS.

The data transmission from the camera present in the simulator and the ROS is performed through the ZMQ communication protocol, which sends the bitmaps images encoded in 32 bits RGBA, which are decoded in the middleware and applied to the location of the mobile robot through the identification of the ArUco fiducial markers. More details about the connection between the ROS and the SimTwo simulation environment and the preliminary tests performed with the localization system using the simulator are available at Braun et al. (2022).

In the real AMR, the UDP and ZMQ communication protocols are not necessary for the integration and exchange of messages between the peripherals and the other components that compose the operational architecture of the robot. This is because it is possible to communicate with the components presented previously in Figure 4 by using serial communication (LiDAR and Arduino) and camera serial interface (RGB Camera). However, the whole system implemented for the control of the robot, as shown in Figure 5, follows the same structure used in the ROS framework developed for SimTwo, with only minor adaptations in the communication with the peripherals through packages inherent to the middleware.

### 3.2 Fiducial markers

As presented earlier in Section 2, fiducial markers are widely present in localization approaches for mobile robots, especially for indoor environments. In the context of the RAF competition, the ArUco fiducial markers mirrored across the factory floor and on the walls of the machines are the primary references for localization in the scenario.

**FIGURE 9:**
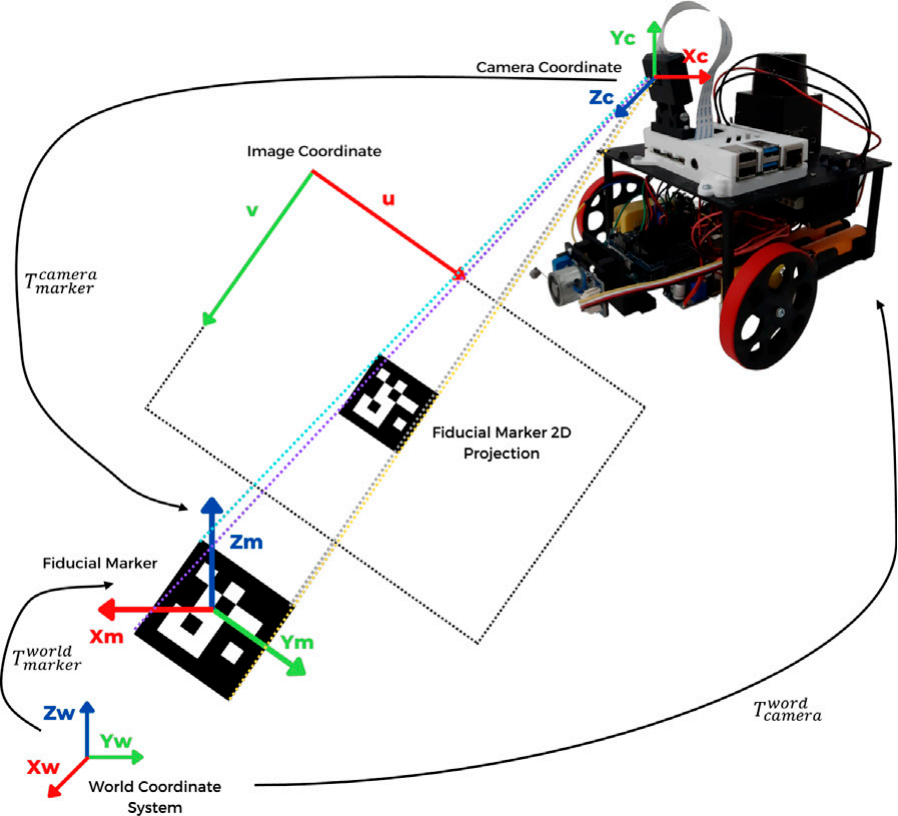
AMR’s pose estimation according to the observation of a fiducial marker.

For the implementation of the localization system presented in this paper, for convenience, the RGB camera module attached to the AMR was adopted as the robot’s reference centre. In the image processing stage for the position estimation of the detected fiducial markers in relation to the camera, the ArUco module from OpenCV Bradski (2000) library was used. Figure 9 represents the process of estimating the AMR’s global position according to the fiducial marker’s observation.

The used ArUco markers have known sizes. Thus, the OpenCV library can determine the distance and orientation between the camera and the tag according to the camera’s intrinsic parameters and the position occupied by the fiducial marker’s centroid in its 2D projection on the image, represented by a point (*u*, *v*), estimated in pixels. Each fiducial marker has a unique identification ID, which the OpenCV library can also decode. In addition, a database in the AMR has stored the absolute pose of each ArUco tag on the competition scene floor, i.e. its position and orientation relative to the scene’s (world) centre. Whenever a marker is detected and has its ID decoded, the algorithm derives the translational and rotational vectors of the marker relative to the camera. Afterwards, the camera’s pose relative to the world is computed, as exemplified by Eq. 2.

$$T_{c}^{w}=T_{m}^{w}\cdot {\left(T_{m}^{c}\right)}^{-1}$$ (2)

Where $T_{m}^{c}$ represents the homogeneous transformation of the fiducial marker frame relative to the camera frame. $T_{m}^{w}$ represents the absolute position of the fiducial marker relative to the world. Finally, $T_{c}^{w}$ represents the camera pose relative to the world.

It is important to note that some markers in an image may not be detected if their characteristics are not identified because the marker can be too distant or even with a very wide angle relative to the camera’s optical centre. Markers identified under these conditions of distance and orientation with a large offset can easily introduce noisy measurements to the localization system. Therefore, the approach presented in the next section with EKF considers these circumstances to reduce the AMR’s localization noise.

## 4 Robot localization

As previously mentioned in Section 1, the RAF competition focus on autonomous mobile robots, their challenges, and the current problems in the industry. Therefore, the contest allows the competitors to use any solution for localization as long as it fits the rules described in Section 2.3. In this case, our minimalist approach for localization, yet effective, take advantage of what the contest provides on the competition floor, fiducial markers.

### 4.1 Position filter

The proposed localization uses an EKF as a basis for the system, and its workflow is described below.

• Prediction phase
  1. State estimation
  2. Filter’s covariance estimation
• Update phase
  1. Innovation
  2. Innovation covariance estimation
  3. Kalman Gain
  4. State estimates update
  5. Filter’s covariance estimate update

**FIGURE 10:**
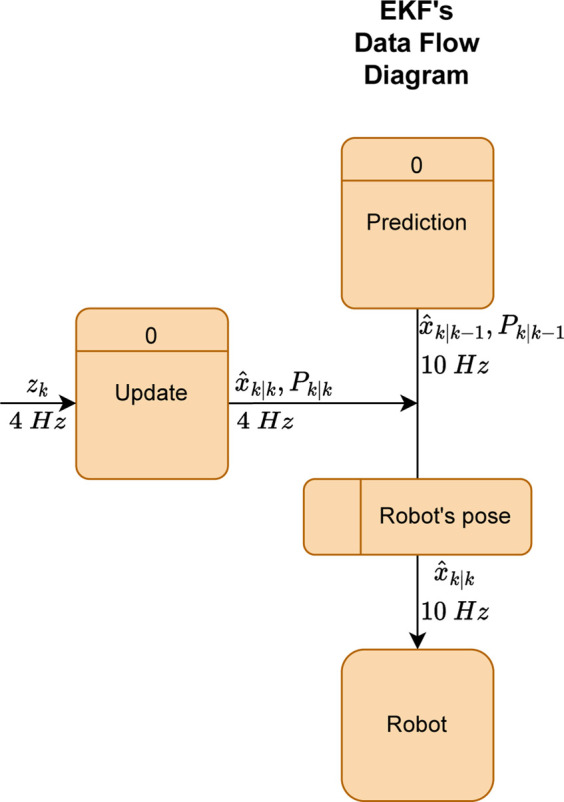
AMR’s EKF-based proposed localization system.

As displayed above, the EKF comprises two phases, prediction and update. In the former stage, the filter makes estimates of the states that are after corrected in the update stage. These two stages do not necessarily happen synchronously. However, if one phase starts, the procedures within are sequential. Moreover, the filter, which runs in discrete time, only moves forward in time whenever a prediction stage happens. In other words, it moves from state *k* − 1 to state *k*. Each procedure in both stages is described throughout this subsection. A data flow diagram (DFD) was made, shown in Figure 10, to explain how the EKF works dynamically in this localization system.

As already mentioned, the DFD depicts the classical structure of an EKF, divided into two stages, prediction and update. As one can see in the figure, the prediction stage is where the process or state transition model, ${\hat{x}}_{k|k-1}$ , and the predicted covariance estimate, ***P***_*k*|*k*−1_, are computed. In this case, the process model is the robot’s odometry, derived from its kinematic model (already presented in Section 3), and the predicted covariance estimate is the filter’s covariance. In other words, the filter’s state estimates are computed based on previous states, as displayed in Eq. 3. The filter’s states after the update stage is represented by ${\hat{x}}_{k|k}$ . Moreover, it is important to emphasize that the robot’s pose states are the filter’s states, updated at a rate of 10 *Hz*, independently if the update stage happens. Finally, ***z***_*k*_ represents the filter’s states after the update stage. Both are going to be described in more detail in this subsection.

$${\hat{x}}_{k|k-1}=f\left({\hat{x}}_{k-1|k-1},u_{k}\right)$$ (3)

As shown in Eq. 3, the new state estimates are computed from the state transition model *f* (***x***, ***u***) that depends on previous estimates and the control vector (***u***_*k*_). In this case, the latter is zero. Eqs 4, 5 represents, respectively, the state transition and predicted covariance estimate models:

$$\left\{\begin{array}{c} x_{k}=x_{k-1}+v_{k-1}\;\cos\left(\theta_{k-1}\right)\;\Delta t\;\\ y_{k}=y_{k-1}+v_{k-1}\;\sin\left(\theta_{k-1}\right)\;\Delta t\;\\ \theta_{k}=\theta_{k-1}+\omega_{k-1}\;\Delta t\; \end{array}\right.$$ (4)

Where [*x*_*k*_, *y*_*k*_, *θ*_*k*_] represents the current robot’s pose state, their [*k* − 1] counterparts represent the previous state, and Δ*t* represents the sampling period. One can note that this process model is not linear, thence the use of the Extended Kalman Filter and not the Kalman Filter. It is important to note the difference between ${\hat{x}}_{k}=\left[{\hat{x}}_{k},{\hat{y}}_{k},{\hat{\theta }}_{k}\right]$ and ***x***_***k***_ = [*x*_*k*_, *y*_*k*_, *θ*_*k*_]. The former represents the filter’s state estimates, i.e., the mean value of the probability distribution of the EKF, which also corresponds to the robot’s pose states. On the other hand, the latter represents the robot’s current states based on the odometry model.

$$P_{k|k-1}=F_{k}P_{k-1|k-1}F_{k}^{T}+Q_{k}$$ (5)

From Eq. 5, ***F***_*k*_ describes the Jacobian of the process model in ${\hat{x}}_{k-1|k-1}$ , usually represented in a state space format. Moreover, ***P***_*k*−1|*k*−1_ represents the previous estimate of the filter’s covariance. Finally, ***Q***_*k*_ is the process noise, i.e., the covariance of the noise of the state transition model, modelled by a Gaussian. The former represents the reliability of the state transition estimates, i.e., a low ***Q***_*k*_ dictates that the estimates are reliable.

The second stage, the update phase, is where the EKF considers exteroceptive sensor data, called observations ***z***_*k*_, into the estimates as innovations. The observations also have a reliability metric modelled by a Gaussian noise ***R***_*k*_, called observation noise, which can be a function that can vary with each observation and iteration of the filter. In this specific case, the exteroceptive sensor that the system uses is the RGB camera, already displayed in Section 3. Finally, the innovations are weighted by the Kalman gain and used to correct the filter states’ estimates. This gain is a variable that depends on the predicted covariance estimate, state transition and observation models, and the process and observation noises. Finally, after correcting the filter states to ${\hat{x}}_{k|k}$ , the covariance estimate is updated to ***P***_*k*|*k*_.

Thereby, as it is possible to see in Figure 10, the prediction phase executes at a rate of 10 *Hz* whereas the update phase occurs at 4 *Hz*. In addition, the robot’s control cycle works in 10 *Hz*, i.e., at the same rate as the robot’s pose update frequency. Finally, as explained at the beginning of this subsection, it is essential to note that the filter advances in “discrete time”, *k*, after making a new prediction. In other words, an infinite number of updates can happen in one state, estimate *k*. In this specific case, for every five state transitions, two updates are performed.

### 4.2 Update stage filters

The Kalman Filter and its non-linear variant, EKF, was developed to generate estimates, using a series of measurements amongst statistical noise and other types of error, that are inclined to be better than estimates that are generated from a single measurement. Even if it can deal with measurement errors, there are other ways to improve its performance. In more detail, two filters were applied in the update stage to verify if the observations were acceptable to insert in the filter as innovations.

To better understand how the filter works, it is elemental the understanding of the dynamics of the EKF. As already shown in Section 4.1, the filter’s covariance keeps diverging as long as only the prediction stage occurs. However, whenever an observation occurs, the covariance starts to converge to a particular value. This dynamic is expected since the process model is a relative estimation method for the robot’s states, whereas the observations are considered absolute state estimation methods. The problem is when wrong observations are inserted into the filter’s process, especially in this case where the observation and process noises were chosen to be constant for all observations and state transitions, respectively. This situation can detrimentally affect the filter performance in two hypotheses. First is when the filter’s covariance estimate matrix is diverged. That is, its eigenvalues are either numbers out of the scope of the system’s normal behaviour, or the system’s filter did not yet converge to a constant value. After wrong observations are considered in the filter, the filter’s covariance eigenvalues will reduce to a particular value, heavily influenced by the observation covariance matrix. In this case, a wrong observation would be detrimental to the filter’s dynamic and, consequently, the localization system. That is to say, not only will the filter’s states tend to converge to a wrong value, but it will also maximize the filter’s belief that the states are correct. Secondly, there is the hypothesis that the filter has already converged to a correct value. In this instance, the different behaviour would be that the covariance matrix would stay converged. However, the innovations would generate noise in the new states, possibly driving them away from the “correct” value. Thus, since the system is designed for an environment with the intent to have several good observations at each update state, it is safe to assume that it is better to ignore a good observation than to accept a wrong one. Thus, one distance filter and a statistical distance filter were implemented, and they are described below.

#### 4.2.1 Euclidean distance filter

As already described in Sections 3.1, 4.1, the observations that are used come only from the ArUco tags. These observations can innovate all the states from the AMR, which is its pose. However, there is a non-linear relationship between the relative orientation (yaw angle) of the position of the camera’s optical centre and the centre of the ArUco tags. That is, depending on the positioning of the camera to the tags, the pose estimation of the robot can improve or worsen. It was perceived that at a fixed distance between the camera and the tag, if the robot started at 0° looking straight into the tag and was rotated, the pose estimation would worsen non-linearly and non-monotonically the more the AMR tended to 90° (the detection would cease before reaching the right angle). In addition, it was also noted that the more the robot was distanced relative to the centre of the tag, with the camera staring straight into it, the more the pose estimations would worsen non-linearly and non-monotonically. Moreover, it is essential to note that this generally happened to all states, namely *x*, *y*, *θ*, however, with more intensity to *θ*. Specifically, at greater distances, *θ* jerked a lot between observations that ideally should be similar. Therefore, a threshold was found experimentally to limit observations above it. In other words, it was verified that observations below the threshold of 0.71 m were acceptable to be inserted in the filter as innovations. In relation to the camera’s orientation to a given tag, it was deemed unnecessary to apply a filter since limiting the observations to closer tags already limits the maximum angle of detection of the tags. In addition, it is important to note that the pitch angle of the camera relative to the robot’s plane, shown in Figure 3, already limits its maximum field of view and, by consequence, the detectable radius.

#### 4.2.2 Mahalanobis distance filter

The Mahalanobis distance measurement is a metric that computes the distance of a point *P* to the mean of a probability distribution *Q* in $R^{N}$ . It considers the data set’s correlations, in contrast to the Euclidean distance, which would assume a spherical data spread. In a more detailed way, the Mahalanobis distance measures the distance of a point *P* relative to the mean of the distribution along each principal component of the covariance matrix of *Q*. Thus, one can note that the Euclidean approach is bad for outlier detection because the data distribution is not spherical, i.e., two observations can have the same Euclidean distance from the mean of the distribution where one can belong to it, following criteria of standard deviations for example, and the other not. In this reasoning, the Mahalanobis distance metric has many applications, one of which is identifying outliers of multivariate observations. Thus, considering a probability distribution *Q* with covariance ***S*** on $R^{N}$ , the Mahalanobis distance from point $y={\left(y_{1},y_{2},\ldots ,y_{N}\right)}^{T}$ to mean $\mu ={\left(\mu_{1},\mu_{2},\ldots ,\mu_{N}\right)}^{T}$ is:

$$d_{Mi}\left(y_{i},Q\right)=\sqrt{{\left(y_{i}-\mu \right)}^{T}S^{-1}\left(y_{i}-\mu \right)}.$$ (6)

In the case of localization, ***μ*** corresponds to ${\hat{x}}_{k|k-1}$ , the mean of the EKF’s states before the update stages. The point ***y*** represents a new observation from exteroceptive sensor data after passing through the observation model that adapts the sensor’s output to the observation space where the filter can perform the innovations. Finally, the covariance ***S*** represents the EKF’s predicted covariance estimate ***P***_*k*|*k*−1_.

Intuitively, one can see that if the principal components of ***S*** were to be reduced to have a variance of one, the Mahalanobis distance would become the Euclidean distance. Thus, testing found that a Mahalanobis distance of 0.08 was sufficient to ignore observations deemed as outliers and keep only the similar ones whilst the AMR was operating. Also, it is important to note that this distance is unitless.

Both filters are displayed in the Pseudocode Algorithm 1.

Algorithm 1 Filter update callback function

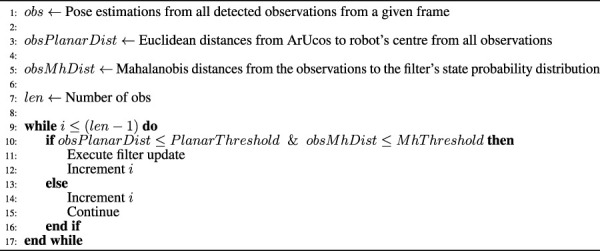

As one can see, this pseudocode generally explains how the filter’s update stage was managed. The pose estimations derived from the detections were already explained in Section 3.1. Finally, this algorithm assumes that every accepted observation was considered individually in the filter and without an order. Although this can be an approach, others were implemented and compared in this work, which is explained below.

### 4.3 Update approach

Following the EKF’s behaviour, as already explained in Section 4.1, one can note several ways the updates can be considered in the filter. In other words, the order and how they are considered impacts the filter’s performance differently, especially by the reasons described in Section 4.2. Although the explanation considers wrong observations, even amongst “good” observations, there are better observations than the others (considering a set of criteria). Considering that several tags can be detected at a given frame of the camera, the question is how to process that information into observations and consider them as innovations in the filter. In this research work, two approaches were implemented and tested. The first one inserts all the accepted observations and performs the updates individually without sorting. The second one sorts all the detected observations following a crescent Euclidean distance criteria. The effect on how the observations are treated in the update stage of the filter is even more potentialized by the Mahalanobis filter. This happens because, as can be seen in Eq. 6, the distance is weighted by the filter’s covariance matrix. On the one hand, if the filter is diverged, the Mahalanobis distances will be low values. This is expected since high covariance values mean high eigenvalues and a wide probability distribution of pose beliefs. On the other hand, if the filter is already converged (low values of ***P***_*k*|*k*−1_), the Mahalanobis distances will tend to be high even with similar pose estimations. Thus, if wrong observations are inserted in the filter, especially if the filter is diverged, the filter will converge, believing that these are the correct states. Therefore, for a period, the Mahalanobis filter will keep rejecting the good observations because these will not belong to the probability distribution according to the set threshold. Only the prediction stage will continue to execute when this happens, and the filter’s covariance will keep increasing its values according to Eq. 5. This translates that the probability distribution covariance will increase in size until it passes the set threshold. After this, the filter will start to accept new observations again. This situation is one of the worst situations that can happen to an autonomous robot since it means that the AMR got lost. Thus, these two approaches were tested to avoid this scenario.

## 5 Results

This section is divided into three subsections. The first addresses and validates the ground truth system used in this work, testing it for accuracy and precision. On the other hand, the second and third subsections test the localization system accuracy, precision, and robustness in scenarios where the robot was kidnapped. In this reasoning, the localization system is tested in all three localization problems, namely position tracking, global positioning, and kidnapped robot problem, since the latter requires the first two to be solved.

**FIGURE 11:**
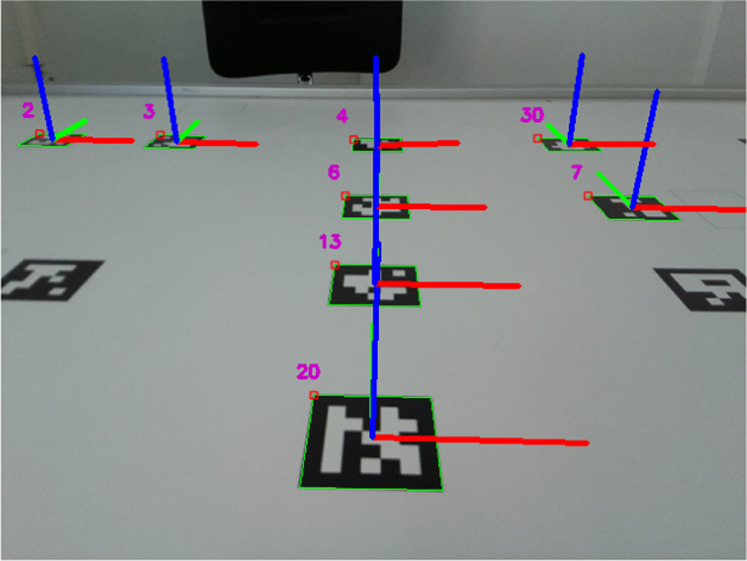
Robot’s camera perspective during the test scenarios.

Still, about the second and third subsections, the robot was positioned directly above ArUco 22, with both centres aligned with an orientation of $\frac{\pi }{2}$ radians. This tag has a global position of [*x*, *y*, *θ*] = [0, − 0.355, *π*]. A camera-based ground truth system was developed to validate the localization system, where it needs two tags aligned with the robot’s centre to estimate the robot’s position. In this reasoning, the ground truth’s estimations can be compared with the robot’s localization system’s estimations. In this way, the setup for the two latter subsections can be seen in Figure 13. Moreover, Figure 11 shows the robot’s camera perspective with the ArUCo tags detected in the test scenarios.

The robot kidnapping was performed digitally, i.e., after the robot was put above tag 22, the mean value of the EKF’s states was jerked to pose [*x*, *y*] = [0.2, − 0.05] in all test cases. The orientation was not changed.

### 5.1 Ground truth

According to the RobotAtFactory 4.0 competition floor, there are 33 ArUco markers with their respective global position and orientation that can be used to perform a ground truth system to validate the proposed localization system of the robot. Thus, to take advantage of these markers, the ground truth system was based on an RGB camera fixed on top of the competition floor. This system localizes all the markers, assuming that the global reference frame is ArUco 13 and that the ArUcos with ID 16 and 4 are the *x* and *y* axes, respectively (refer to Figure 3 for better visualization). Therefore, it is possible to estimate the position and orientation of all the other markers after calibrating the camera’s intrinsic parameters and knowing the coordinates of these three markers. For better precision in the orientation estimation, it was chosen to compute the pose using two markers. Consequently, two markers were placed beside the robot’s wheels aligned with the robot’s centre so that the middle point between the two ArUCos becomes the robot’s centre. In other words, computing the position of these two markers is possible to obtain the robot’s centre (average) and its orientation (*atan*2 function). The details of this ground truth system are not the focus of this paper, but its accuracy and precision were observed, and they are presented as follows.

**FIGURE 12:**
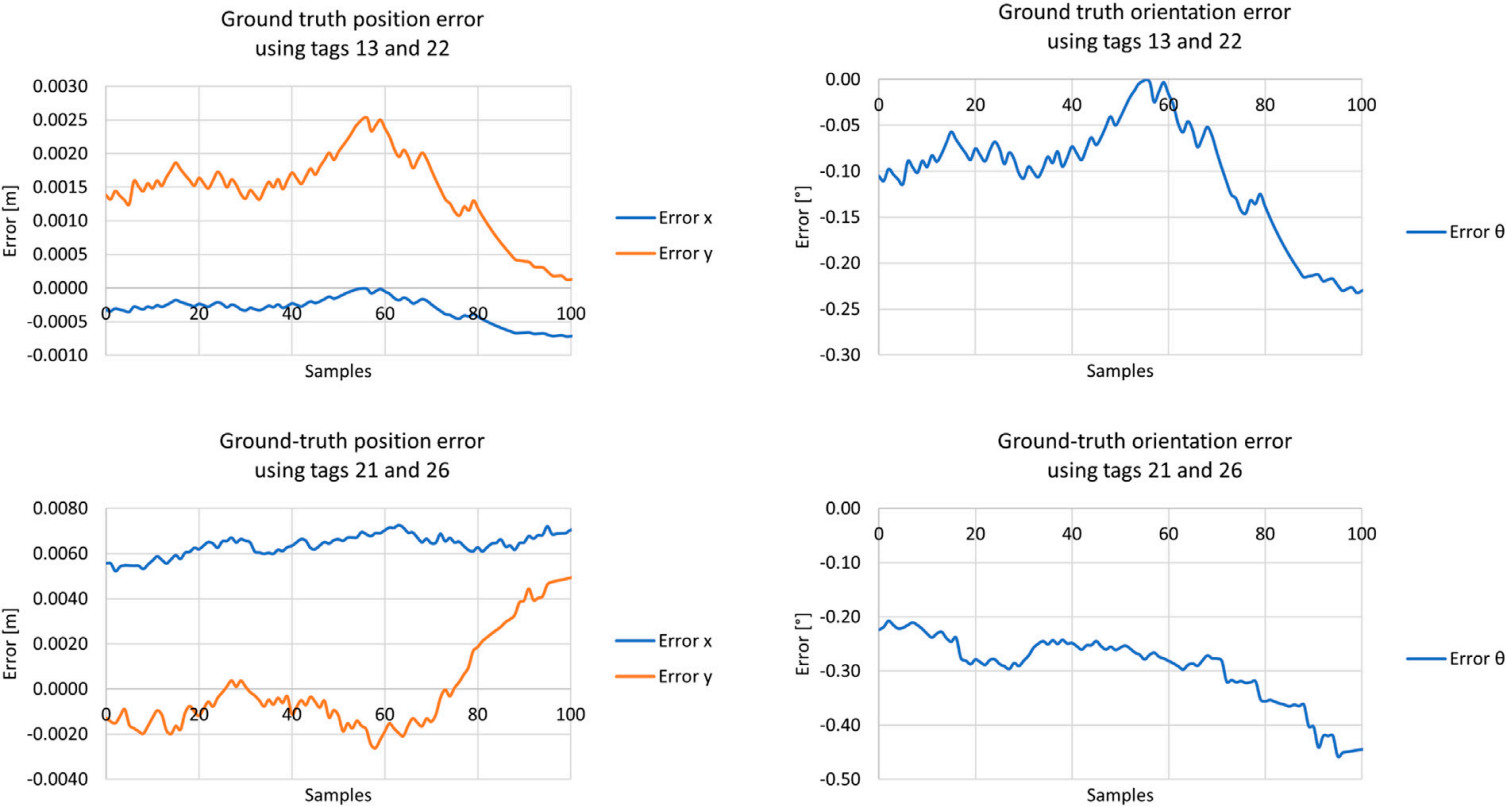
Ground truth system validation using tags 13 and 22 (first row), and 21 and 26 (second row).

As the camera from the ground truth system is positioned on top of the competition floor, there are numerous possibilities to validate the ground truth system. Moreover, since it is a camera-based system, it has several types of distortion. Therefore, two scenarios were chosen to present in this article, considering they were areas near the scene where the AMR’s localization system was tested. In this reasoning, the system’s behaviour shown in these two tests better represents its accuracy and precision when used as a reference for the localization system. Thus, one can see in Figure 12 the position and orientation error from the middle point between the pairs of the ArUcos 13 and 22, and 21 and 26. Both scenarios’ root mean square errors (RMSE) were computed for accuracy and are presented in Table 1. In both scenarios, the ground truth system presented millimetric error in *x* and *y* and fractional error in orientation.

**TABLE 1:** Root-mean-square error from the ground truth’s pose tests.

Tags	RMSE x [m]	RMSE y [m]	RMSE *θ* [°]
13 and 22	0.0005	0.0014	0.1470
21 and 26	0.0064	0.0024	0.3169

The standard deviations of each state were also computed to visualize the system’s precision, and they are presented in Table 2. In both scenarios, it is possible to observe that the system is precise, showing slight deviations in all states.

**TABLE 2:** Standard deviations from ground truth’s pose tests.

Tags	Std. x [m]	Std. y [m]	Std. *θ* [°]
13 and 22	0.0002	0.0007	0.0743
21 and 26	0.0004	0.0023	0.0679

**FIGURE 13:**
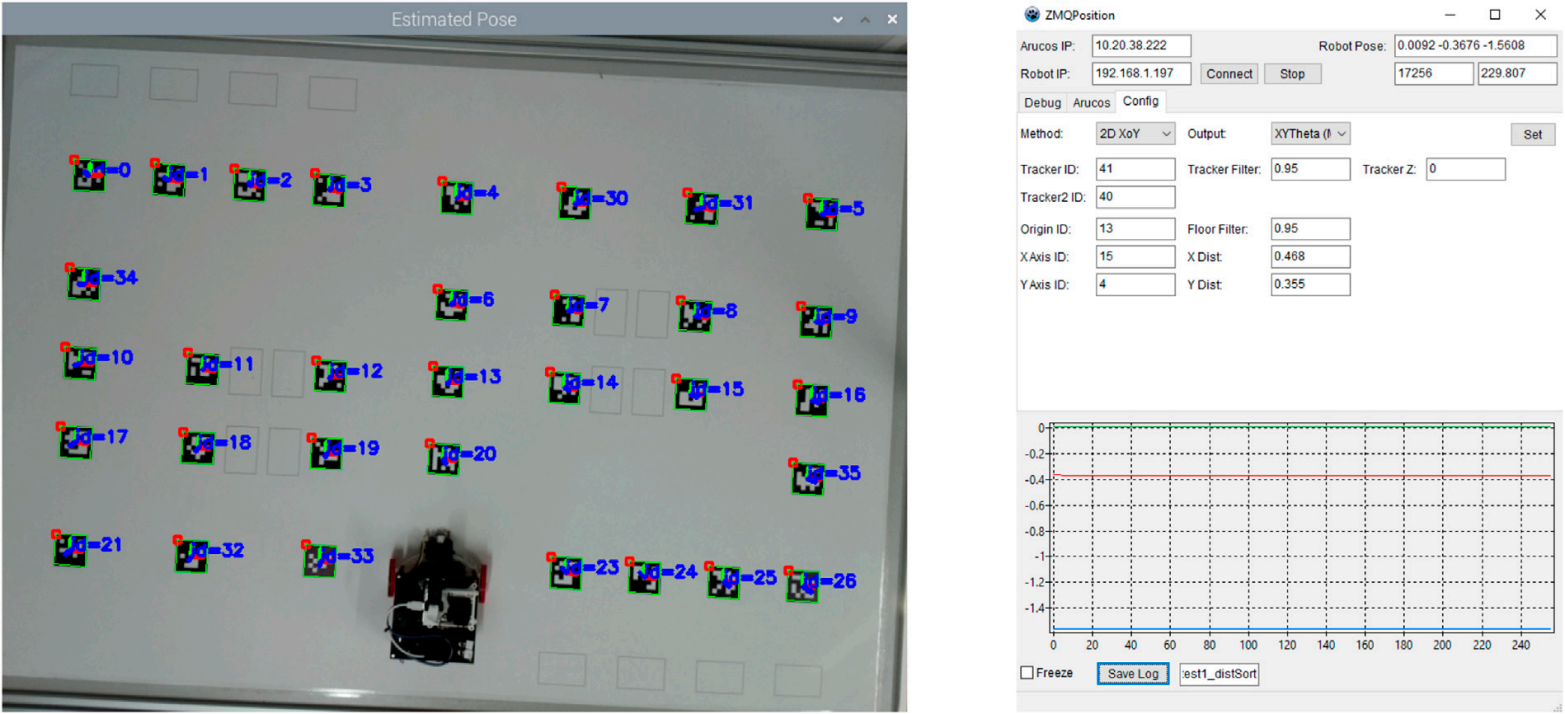
Ground truth system’s perspective (left). Ground truth graphical application (right).

Finally, a picture of the ground truth system’s vision perspective and its application can be visualized respectively in the left and right pictures of Figure 13.

### 5.2 Kidnapped robot problem with mahalanobis filter

Two scenarios were tested with Mahalanobis and Euclidean distance filters enabled. They differ regarding the innovation approach, i.e., how the observations were inserted in the filter.

#### 5.2.1 Unsorted observations

The first scenario inserts the observations as innovations without proper sorting. In other words, they come in the order the OpenCV library detects the tags.

**FIGURE 14:**
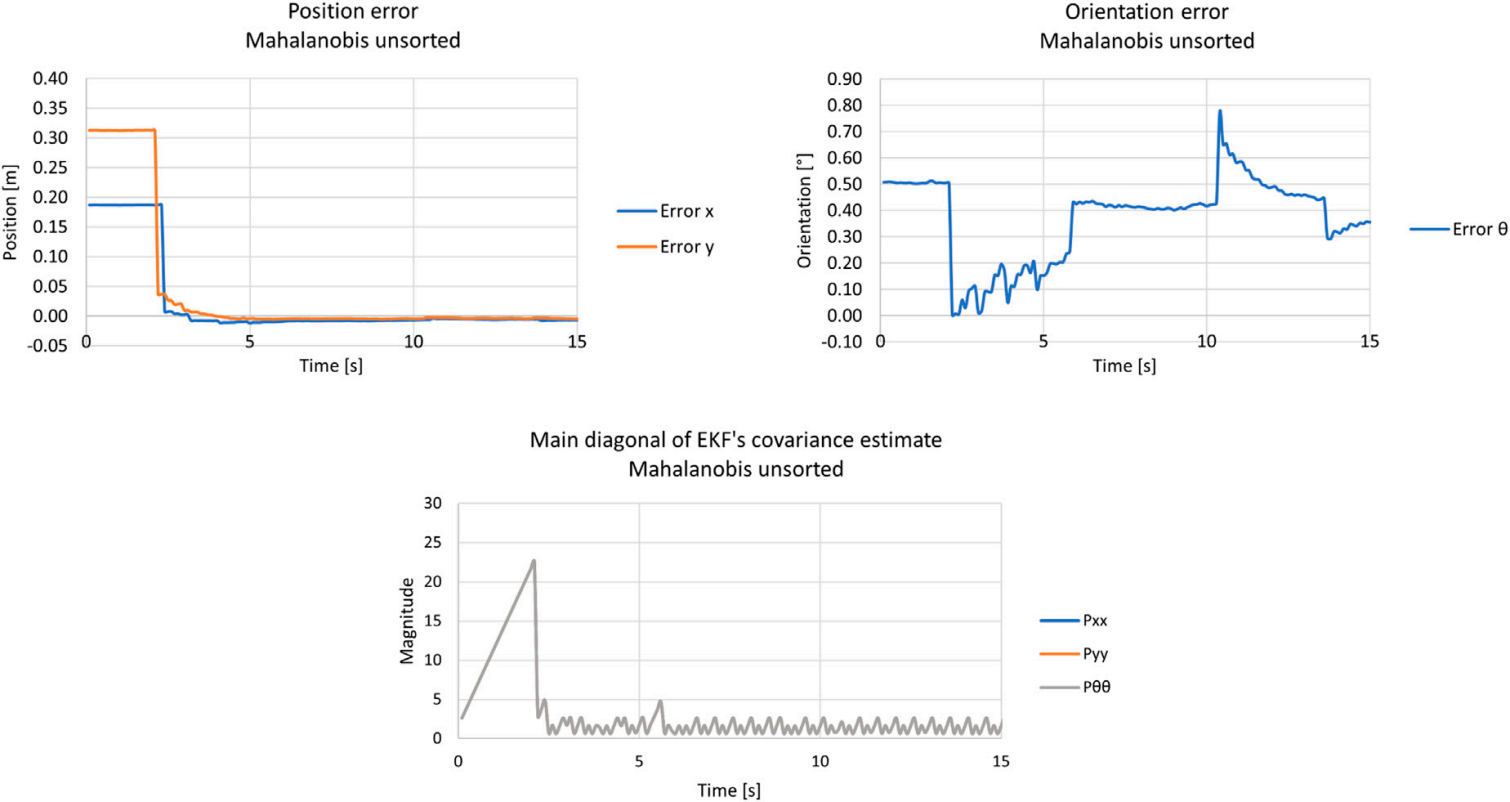
Results from the kidnapped robot test with the unsorted approach and Mahalanobis filter. The Upper left graph represents the position error, the upper right displays the orientation error, and the bottom graph shows the main diagonal of the EKF’s covariance estimate.

It was chosen to plot only the main diagonal of the filter’s covariance matrix since it was assumed that the filter’s states were not correlated. In this reasoning, one can see in bottom graph of Figure 14 that the filter’s main diagonal covariance increases linearly until it surpasses the ellipse created by the Mahalanobis Filter. The increase is linear since the robot was not moving. Soon after this threshold is met, the covariance decreases drastically because the EKF starts to accept observations as innovations. The intensity in the decrement of the covariance matrix is directly correlated with its magnitude when the threshold is met, i.e., the more the covariance increases, the greater the decrement. Consequently, when the covariance decreases, it means that the filter converged to a mean value, and this can be seen in the left and right graphs of Figure 14, where the left plot shows the position error decreasing instantly, and the right displays a drastic change in orientation. It is important to note that the Mahalanobis filter makes the EKF’s pose estimate hold the last value before the kidnapping until it surpasses the chosen threshold, i.e., the Mahalanobis filter highlights the kidnapping behaviour.

#### 5.2.2 Distance-sorted observations

In this scenario, the test was also performed with the same setup as the previous one, with distance-sorted observations. In more detail, the observations were sorted in a crescent manner by the planar distance between the centre of each ArUco tag and the camera’s optical centre.

**FIGURE 15:**
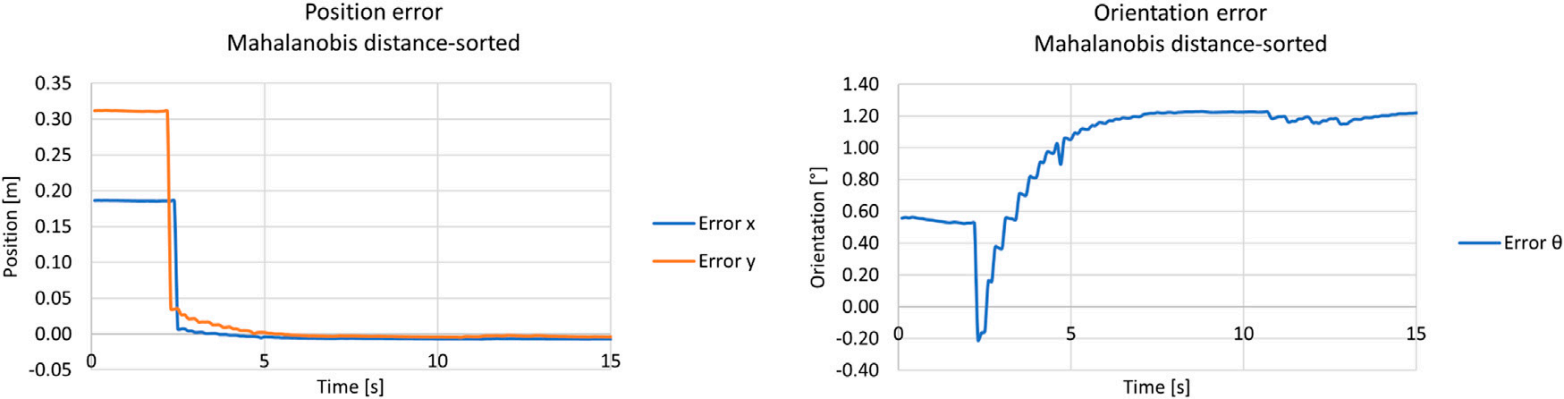
Results from the kidnapped robot test with the distance-sort approach and Mahalanobis filter. The left graph shows the position error, whereas the right shows the orientation error.

The covariance graph was not shown for space purposes since it has similar behaviour to the unsort approach, increasingly linearly until it reaches the Mahalanobis filter threshold. The position and orientation error can be seen in the left and right graphs of Figure 15, respectively. Both graphs show a stable convergence to a mean value and have similar behaviour as the previously unsort approach with Mahalanobis.

In addition, same as the ground truth system, standard deviations and RMSE were computed for each scenario and each approach for comparison. The difference is that the statistics were divided into transient and permanent stages of the convergence of the filter since it better represents the filter’s behaviour during the comparison. Thus, the standard deviations for the presented tests, for the transient and permanent regimes, can be seen in Tables 3, 4, respectively, whereas Table 5 describes the RMSE computations.

**TABLE 3:** Standard deviations from the localization system’s kidnapped robot tests—transient.

Innovation approach	Std. x [m]	Std. y [m]	Std. *θ* [°]
Unsorted	0.054	0.078	0.15
Dist. sort	0.036	0.056	0.26
Unsorted + Mahalanobis	0.081	0.130	0.17
Dist. sort + Mahalanobis	0.092	0.150	0.24

**TABLE 4:** Standard deviations from the localization system’s kidnapped robot tests—permanent.

Innovation approach	Std. x [m]	Std. y [m]	Std. ***θ*** [°]
Unsorted	0.0017	0.0032	0.061
Dist. sort	0.00064	0.00045	0.062
Unsorted + Mahalanobis	0.00086	0.00060	0.099
Dist. sort + Mahalanobis	0.0011	0.0022	0.067

**TABLE 5:** RMSE from the localization system’s kidnapped robot tests—permanent.

Innovation approach	RMSE x [m]	RMSE y [m]	RMSE *θ* [°]
Unsorted	0.006	0.008	0.301
Dist. sort	0.003	0.009	1.803
Unsorted + Mahalanobis	0.006	0.004	0.465
Dist. sort + Mahalanobis	0.007	0.004	1.319

### 5.3 Kidnapped robot problem without mahalanobis

The same two scenarios were tested with the Mahalanobis Filter disabled, and the euclidean distance filter enabled. Both are described below.

#### 5.3.1 Unsorted observations

**FIGURE 16:**
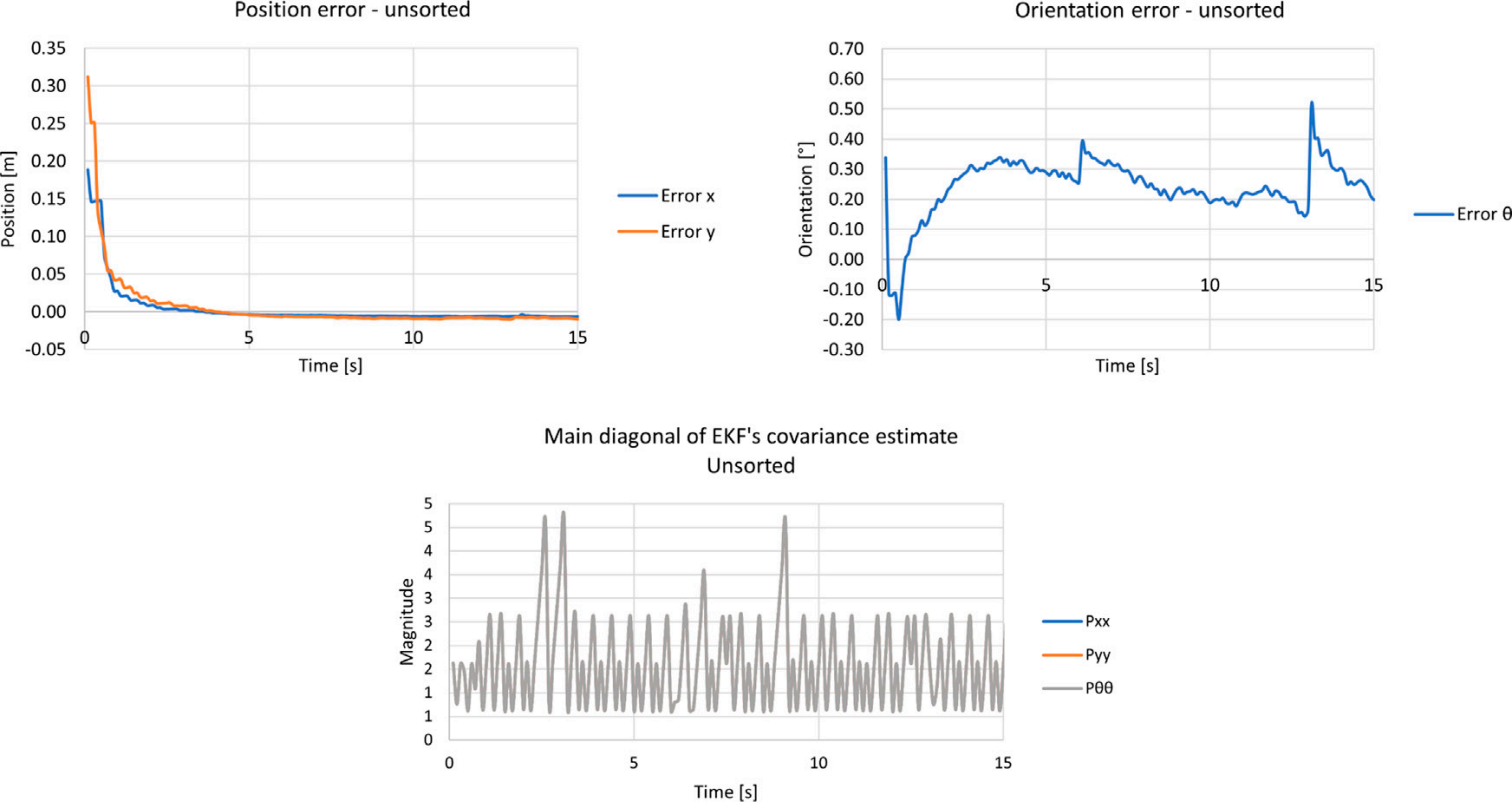
Results from the kidnapped robot test with the unsort approach and without Mahalanobis filter. The upper left graph displays the position error, the upper right, the orientation error, and the bottom graph shows the main diagonal of the EKF’s covariance estimate.

The first scenario was tested with the unsorted approach. In this reasoning, the position error, the orientation error, and the main diagonal of the filter’s covariance can be seen in the upper left, upper right, and bottom graphs of Figure 16, respectively.

As shown in Figure 16, the filter’s behaviour is entirely different. This is expected since the Mahalanobis filter was not enabled and did not influence the kidnapping. Therefore, the filter did not diverge after the kidnapping since new observations entered the filter as if the robot was in the new position. The orientation and position errors during the kidnapping in this approach converged without having the period where the EKF holds the last value until it needs to surpass the Mahalanobis threshold.

#### 5.3.2 Distance-sorted observations

**FIGURE 17:**
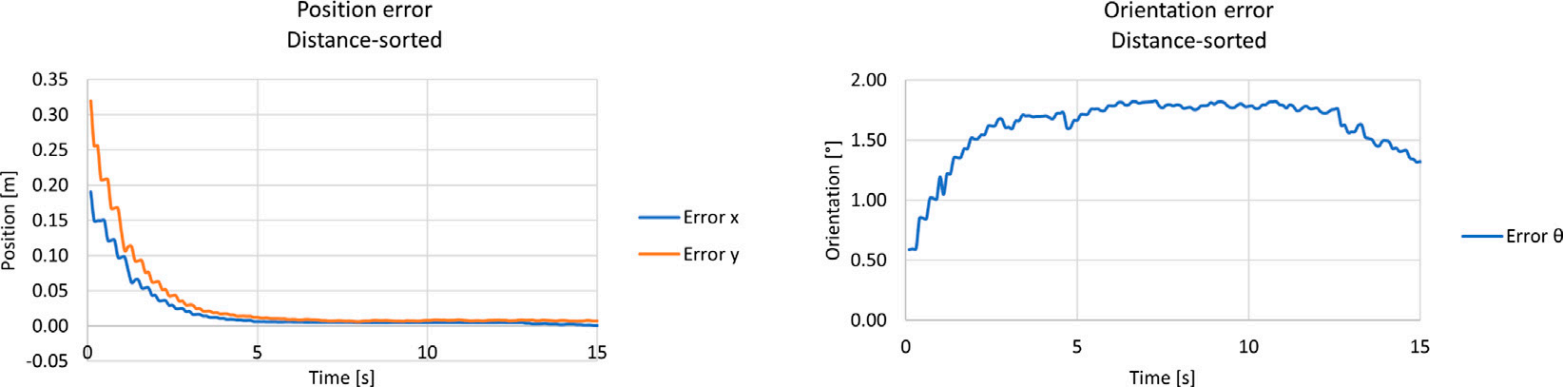
Results from the kidnapped robot test with the distance-sort approach and without Mahalanobis filter. The left graph shows the position error, and the right graph the orientation error.

The distance-sorted approach was also tested without the Mahalanobis Filter. The covariance matrix from the filter was also omitted since it has similar results to the previous scenario. The position and orientation errors can be seen in the left and right pictures of Figure 17, respectively.

As shown in the figures above, the approach had similar results as the previous scenario, showing a steady convergence to a mean value of the filter’s states. Furthermore, same as the scenario with the Mahalanobis Filter enabled, standard deviations and RMSE were computed for each state of the filter and each approach for comparison, and they are presented below.

Table 3 displays the standard deviations in the transient state of the EKF. One can see that the innovation approaches did not show a significant difference between them. This can be explained since these tests did not stress the system enough. In other words, these tests presented only good observations. In future work, tags with noisy detection must be inserted to highlight the differences between these approaches. Moreover, comparing the results with and without the Mahalanobis filter, one can conclude that this result is expected since this filter’s main advantage and objective is to reject bad observations. However, in the kidnapped robot problem, the EKF is tricked into thinking it is in a different place. In other words, the mean value of the filter’s state is changed, but the robot is not. Thus, the Mahalanobis filter rejects the good observations and the filter, consequently, diverges, and their statistics computations (standard deviation and RMSE) increase. However, it is essential to note that this result depends on the distance of the kidnapping and the Mahalanobis threshold.

Table 4 shows the standard deviations from the permanent state of the filter from the approaches tested in each case scenario. Thus, these deviations are computed after the filter reaches the permanent regime with criteria of 2% of the original value. As one can see, after the filter’s convergence, the standard deviations significantly decrease for all approaches, and no significant difference is notable between them.

Table 5 presents the RMSE computed during the permanent regime of the filter’s convergence since the transient analysis from the perspective of RMSE does not add value to the work. This happens because the results would be influenced by the Mahalanobis filter and the position of the kidnapping. Therefore, RMSE could be lower or higher depending on how distant the kidnapping is from the original position, producing an unfair comparison to the approaches without the Mahalanobis filter.

Observing Table 5, it is possible to see that the localization system is adequate despite the approach used, presenting an error of less than 1 cm in *x* and *y* states and less than 1° in *θ* for the unsorted approaches and less than 2° in the distance-sorted approaches. The only significant difference between the approaches is that the distance-sorted approach presents a worse performance in the orientation error compared to the unsorted approach. Since the robot is small, the radius of operation of the robot is small. Thus, an error of less than 2° is good enough for this application.

## 6 Conclusion and future work

This paper proposed a localization system based on ArUco markers and EKF that allow the AMR to navigate within the RAF competition. Moreover, a ground truth system was developed and used to validate the localization system, allowing a measurement of the robot’s pose with high precision and accuracy. Two innovation approaches were implemented and tested. The first method inserts all the accepted observations and performs updates individually without a specific order, and the second sorts all the detected observations following a crescent Euclidean distance criteria. The results showed that in these tests, both approaches showed negligible differences. The tests were performed following the kidnapped robot problem scenario because this problem considers both the position tracking and the global positioning problem. Despite the innovation approach, the robot’s localization system presented high precision and accuracy, validating that this system is adequate to operate in this competition. In addition, the Mahalanobis filter was used and tested in the EKF to reject bad observations. It was concluded that it emphasizes the impact of the kidnapping problem on the robot, and since there were only adequate observations, its effectiveness was not assessed.

In future work, further testing must be performed to stress the position filter enough, highlighting the differences between both innovation approaches and the Mahalanobis filter effect on rejecting outliers. A possible case study for testing is the robot navigating through the competition floor whilst it measures ArUcos with noisy data (bad observations) and, in this way, forcing the position filter with the different approaches and filters to present different results. Moreover, it is intended to try to model the non-linear relationships between the relative position and orientation of the camera and the ArUco tags into covariance matrices to better represent the observation noise in the EKF. Finally, to increase the robustness and redundancy of the system, add other types of exteroceptive sensors to the system, such as LiDAR with beacons or natural landmarks, and afterwards, compare the developed methodology with other state-of-the-art approaches.

## Data Availability

The raw data supporting the conclusion of this article will be made available by the authors, without undue reservation.

## Footnotes

1
https://github.com/P33a/SimTwo.

2
https://zeromq.org/.
